# Enhancing the Wellbeing of Family Dementia Caregivers: The Impact of Gamification in the MapHabit Program

**DOI:** 10.1002/alz.088558

**Published:** 2025-01-09

**Authors:** Janelle Gore, Brittany Montgomery

**Affiliations:** ^1^ MapHabit, Atlanta, GA USA

## Abstract

**Background:**

The number of caregivers providing care for a family member or friend is on the rise. Dementia caregivers experience higher levels of stress and burden than caregivers of other chronic diseases due to the physical and emotional demands, and long duration of care provided. Thus, innovative tools are needed to aid in reducing caregiver stress and burden. MapHabit, is a novel mobile health assistive technology tool designed to support families affected by dementia by providing daily schedules and step‐by‐step visual routines and cues called maps, aiding the person living with dementia (PLWD) to complete various activities of daily living (ADLs). With repeated use of these maps, persons with memory impairment have reported an improvement in memory and caregivers have reported reduced stress and burden. Gamified features were integrated into MapHabit to evaluate whether cognitive engagement activities could improve an individual’s motivation to consistently engage with MapHabit. Building upon the demonstrated impact of MapHabit, this study explores the effects of implementing gamified features within the application to enhance the reduction of caregiver stress.

**Methods:**

A modified Zarit Burden Interview was used to assess caregiver burden. The Mini Mental State Examination was used to evaluate the cognitive function of the PLWD. Scores from the respective assessments were calculated based on the respective scale’s scoring guide for pre‐ and post assessments. Scores were then compared to determine the stress and an improvement in memory of the PLWD.

**Results:**

Thirteen participants engaged in the MapHabit with Gamification group for 6‐weeks. About 69% of caregivers reported a reduction in stress and burden post study and 69% of PLWD reported an improvement in memory. Preliminary findings suggest the introduction of gamification in the MapHabit program may aid in reducing caregiver stress and burden.

**Conclusion:**

This research has implications for the ongoing development of dementia support programs, emphasizing the potential of gamification as a supplementary tool to enhance the engagement of existing interventions supporting families affected by dementia.

